# Profiles of Early Childhood Adversity in an Urban Pediatric Clinic: Implications for Pediatric Primary Care

**DOI:** 10.3390/children10061023

**Published:** 2023-06-07

**Authors:** Sarah Ronis, Katherine L. Guyon-Harris, Kimberly Burkhart, Mary Gabriel, Kristin Cipolla, Jessica L. Riggs, Alissa Huth-Bocks

**Affiliations:** 1 UH Rainbow Center for Child Health and Policy, Department of Pediatrics, University Hospitals Rainbow Babies & Children’s Hospital, Cleveland, OH 44106, USA; sarah.ronis@uhhospitals.org; 2 Department of Pediatrics, University of Pittsburgh School of Medicine, Pittsburgh, PA 15213, USA; kag245@pitt.edu; 3 Department of Pediatrics, University Hospitals Rainbow Babies & Children’s Hospital, Cleveland, OH 44106, USA; kimberly.burkhart@uhhospitals.org; 4 Department of Psychiatry, University Hospitals Cleveland Medical Center, Cleveland, OH 44106, USA; mary.gabriel@uhhospitals.org; 5 Department of Pediatrics, University Hospitals Cleveland Medical Center, Cleveland, OH 44106, USA; kristin.cipolla@uhhospitals.org; 6 Department of Psychiatry, Michigan Medicine, University of Michigan, Ann Arbor, MI 48109, USA; jlriggs@med.umich.edu; 7 Merrill Palmer Skillman Institute, Division of Research, Wayne State University, Detroit, MI 48202, USA

**Keywords:** screening, early childhood adversity, primary care, pediatrics

## Abstract

Pediatricians are well-positioned to screen for early childhood adversities, but effective responses to positive screens require an understanding of which adversities typically co-occur, and to what extent they are associated with other risk or protective factors. Among children seen at an urban academic pediatric practice, this study aimed to (1) examine the prevalence of different types of early adversity and protective experiences reported by primary caregivers, and (2) define latent classes of co-occurring adversities. Of 1434 children whose parents completed the Safe Environment for Every Kid (SEEK) at well-child visits during November 2019–January 2021, three classes of adverse experiences emerged, including those reporting low adversity (L; 73%), caregiver stress (CS; 17%), and both caregiver stress and depression (CSD; 10%). Among those who also completed the Adverse Childhood Experiences Questionnaire (ACE-Q, *n* = 1373) and the Protective and Compensatory Experiences Scale (PACES, *n* = 1377), belonging to the L class was associated with lower ACE-Q and higher PACES scores. For parent-respondents only, ACE-Q scores were significantly greater for the CSD class compared to the CS and L classes. Pediatricians should attend to the needs of caregivers reporting both stress and depression, as these families may face especially high levels of adversity and low levels of protective factors.

## 1. Introduction

Childhood exposure to traumatic events has been broadly recognized as a major public health problem in the United States [1,2,3]. Early life adversities have the most devastating impacts due to sometimes irreversible changes in neurodevelopment [4,5,6]. Despite the overall high prevalence of early childhood adversity and the concerning short- and long-term effects on development and health [7,8,9], accumulating evidence shows that certain protective factors may reduce the likelihood of trauma occurrence, as well as negative outcomes following exposure to adversity [10,11,12,13]. “Protective factors” are influences that facilitate adaptive functioning and resilience and help buffer the effects of stress and adversity; resilience, in turn, is thought to be dynamic and multidimensional and assists individuals with healthy living and wellness so that they thrive [14]. Resilience among adults contributes to positive parenting and better outcomes for their own children [15]. Although focusing on promoting resilience within an individual is vital, it is important to also attend to broad societal and systemic factors that create the need for resiliency in the first place, particularly since these factors are experienced by racial and/or ethnic minority groups, economically disadvantaged, and lower-resource communities at greater rates in the United States [16,17,18]. These factors in and of themselves become social determinants of health, leaving some communities at greater risk of experiencing adversity, and with fewer resources dedicated to combat said adversity.

Pediatricians are often tasked with identifying children and families who may need additional support to combat experiences of adversity [19,20,21]. Far and away, the most oft-cited and strongest predictor of resilience for children is the availability of close, protective, and nurturing relationships with parents and other primary caregivers [3]. Building safe, supportive, and nurturing parent–child relationships is endorsed by the American Academy of Pediatrics (AAP, DuPage County, IL, USA) as a key priority for pediatricians and pediatric primary care practices [13,22]. Often referred to as Early Relational Health (ERH), these positive, early, bidirectional relationships between young children and their parents and/or caregivers are posited to buffer the effects of adversity on child development and have the potential to disrupt problematic development via supportive and responsive relationships [13,23].

Despite recommendations that pediatricians screen for adverse childhood experiences (ACEs) and implement strategies to promote safe, stable, nurturing relationships within those families determined to be at the highest risk [24,25,26,27], most of the literature on pediatric populations has focused on outcomes associated with overall load (or count) of ACEs across childhood, e.g., 4 or more vs. fewer than 4 adversities [7,27,28]. Limitations of a count-based approach for clinical practices include the potential equal weighting of widely disparate risks (e.g., food insecurity and parental depression) that likely require different prevention and intervention strategies [29,30]. Indeed, the emerging literature from the child trauma field indicates that the type of trauma matters for specific developmental outcomes [31,32], although, with the exception of child abuse and neglect, little is known about the prevalence and co-occurrence of specific adversities in the earliest years [33].

Furthermore, few studies have examined rates and co-occurrence of both adversity and family strengths within the same study. Recent calls within the pediatric community have noted the importance of focusing on family strengths and resilience rather than applying a purely deficit or problem-focused perspective [13,22,34]. As such, ERH approaches aim to align with families to identify and promote positive experiences within families, while working to address areas of concern [12,13]. For pediatricians seeking to design and implement practice-based responses to adversity, an understanding of which types of adversities most frequently co-occur may guide the selection of response strategies, for example, by helping practices to empirically define eligibility criteria for participation in secondary prevention or intervention programs [35]. In turn, understanding associations between different groupings of adversities and levels of protective factors reported by families may highlight how family strengths can be leveraged to mitigate the effects of adversity on child outcomes [36]. This novel approach to pediatric care stands in contrast to the traditional problem-focused model of care.

This study examined caregiver-reported family adversities, including those known to be associated with child maltreatment [37], among a large sample of low-income families with young children (birth to 6 years) being seen for routine pediatric care at an urban health clinic. Using latent class analysis, we examined whether certain types of family adversities cluster together to reveal meaningful groups within the larger sample, a type of analysis not yet explored with items on the Safe Environment for Every Kid (SEEK, Baltimore, MD, USA), our measure of family adversity. We also explored whether counts of child-specific adverse experiences and protective factors differed based on clusters of SEEK adversities. It was expected that adversity clusters, or groups, would differ on a total load of child-specific adversity and a total load of protective factors, but specific hypotheses were not made, as adversity groupings were identified through data-driven methods.

## 2. Materials and Methods

Participants were caregivers of 1434 unique pediatric patients (0–6 years old; 51.4% female) seen at a high-volume (>13,000 unique pediatric patient visits per year), urban primary care pediatric practice affiliated with an academic children’s hospital in the Midwest region of the United States. Caregivers were mostly (72.6%) parents; however, the remaining (27.4%) consisted of aunts/uncles, grandparents, and biologically unrelated primary caregivers. Slightly over half of those screened for early adversity were 0–3 years of age (55.9%), while 44.1% were 3–6 years of age.

Detailed demographic data are not available for the specific sample included in this report as only child age, child biological sex, and caregiver type were on the primary screening instrument; however, they are believed to represent the general patient population at the clinic, where patients identify primarily as Black or African American (>94%) and are largely Medicaid-eligible (>85%). Over 70% of pediatric patients seen at the clinic reside in the neighborhoods directly surrounding the clinic located in Cleveland, Ohio, which make up the city’s most impoverished historically red-lined neighborhoods. About 20% of patients live in first-ring suburbs, and the remainder lives throughout the Northeast area of Ohio.

As part of regular clinical care, caregivers accompanying young children ages 6 months to 6 years to well childcare visits were asked to complete several screeners addressing social determinants of health, adversity, and family protective factors. Paper screens were presented to caregivers upon check-in and rooming by clinic staff and completed on their own, either in the waiting room or the clinic exam room before the pediatric provider came in. Screeners took a maximum of 15 min to complete. Screens included a cover sheet explaining the purpose of the screeners as follows:

Dear Parent/Caregiver/Guardian,

At the Rainbow Center for Women & Children we care about the total health and wellness of every child and family we serve. As part of our wellness program we ask that a variety of screenings be completed so that our providers have a better understanding of how we can support our patients. We have added screenings about life stress, as well as about family strengths and supports. The forms ask some personal questions; we will keep this confidential among our healthcare team. The purpose of these screenings is to help us better support you and your child. Thank you for partnering with us during your child’s/children’s care.

Caregivers gave completed screeners to their pediatric provider(s) during the visit; providers discussed responses with patients and collaborated on any needed follow-up supports. Screeners were scanned and uploaded to the patient’s electronic medical records.

For the current study, responses on screeners collected between November 2019 (when these particular screeners were first implemented in the pediatric clinic) and January 2021 were utilized; data included screens collected one time per child. This study was reviewed and approved as a chart review study by the University Hospitals Cleveland Medical Center Institutional Review Board (IRB).

### 2.1. Measures

Safe Environment for Every Kid (SEEK; [35,38,39,40]): The SEEK is a 16-item questionnaire assessing the presence/absence of various adversities within the immediate family designed for use within primary care settings. In addition to individual items asking about the desire to have the number to local poison control and the availability of a smoke detector in the home, the remaining items are grouped into six domains with two items each: food insecurity, harsh parenting, major stress, caregiver depression, intimate partner violence, and substance use. The presence of any of these six domains is indicated by a “yes” answer to any of the items tapping that particular domain (e.g., yes to either of the two items about food insecurity). Presence–absence of these six domains were used for the latent class analysis. Extensive research has demonstrated that the SEEK is acceptable and feasible to clinicians and helps to reduce child maltreatment, supporting its validity in pediatric primary care [35,38,39,40].

Adverse Childhood Experiences Questionnaire (ACE-Q; [7]): The ACE-Q is a screener used to measure childhood exposure to ACEs since birth. Caregivers were instructed to look at a list of 17 ACEs and indicate the total number experienced without indicating which particular ACEs had been experienced (i.e., we utilized the ‘de-identified’ version). In addition to the 10 original ACEs, which measure three types of abuse, two types of neglect, and five types of ‘household dysfunction’, the ACE-Q includes seven additional adversities known to affect child health and development including, for example, exposure to bullying, changes in caregiving environment, and experiences of racism. The ACE-Q has been widely adopted as a screener in healthcare settings. Evidence for the tool’s validity comes from several studies demonstrating associations between the original ACEs and the ACE-Q with other measures of maltreatment and with child social–emotional–behavioral outcomes [7,41].

Protective and Compensatory Experiences Scale (PACES; [42]): The PACES is a 10-item measure of the presence/absence of various protective factors that were originally designed for school-age children and older children and their families. Evidence for reliability and validity has been reported [43]. Because our screeners were aimed at identifying adversity and strengths in early childhood, our team modified the measure to include age-appropriate protective factors based on the existing early childhood literature. Specifically, items asked about child assets/internal strengths, availability of consistent and protective caregivers, community connectedness and safety, and family/home order (absence of chaos). Like the ACE-Q, in our clinic, caregivers were asked to look at the list of protective factors and indicate a total score that was true for their child/family.

### 2.2. Data Analysis

Prevalence data for adversities represented on the SEEK were described by the number and percent of caregivers who endorsed each item to characterize the sample. Heterogeneity in SEEK endorsement was analyzed using latent class analysis (LCA). LCA identifies the presence (or absence) of underlying individual differences within a sample which allows researchers to draw conclusions about meaningful, homogeneous subsets or groups of participants within a heterogeneous sample [44]. Analytic techniques such as LCA are referred to as “person-centered” because they highlight individual differences that are often missed in more traditional variable-centered approaches such as correlation and regression, which is a significant strength when seeking to identify how different types of traumatic experiences cluster together. On the other hand, variable-centered techniques assume homogeneity within a sample, meaning that important individual differences go undetected and unappreciated [45]. Compared to other grouping methods such as cluster analysis, LCA is preferred as a more flexible and sophisticated approach that allows for confirmatory, between-groups analysis, a wider array of fit indices, and it considers wider properties of the data such as the distribution of the data. A 2-class model was first tested to establish the presence of heterogeneity in experiences. Next, models for additional classes were tested up to a 5-class solution. Model fit was evaluated using the bootstrap likelihood ration test (BLRT), Lo–Mendel–Rubin likelihood ratio test (adjusted), Bayesian information criteria (BIC), and Akaike information criteria (AIC), which are empirically supported fit indices [46]. Statistically significant (*p* < 0.05) BLRT and LMR-LRT adjusted tests and lower BIC and AIC values indicate good model fit. Entropy and posterior probabilities, which are indicators of model classification quality, and interpretability of the classes were also considered in determinations of the best fitting model. All LPA analyses were completed in Mplus with MLR for missing data. Mean differences between classes in the best-fitting model and scores on the ACE-Q and PACES were analyzed in a multivariate generalized linear model (GLM) framework in SPSS with exported class membership from Mplus. Effect sizes for each model are provided.

## 3. Results

Sample characteristics and SEEK item endorsement are reported in Table 1. Request for the poison control number was the most frequently endorsed SEEK item followed by wishing one had more help with their child, having a smoker in the home, extreme caregiver stress, and items related to food insecurity. Regarding endorsement within each of the six categories used in the latent class analysis, 16% of caregivers reported food insecurity, 4.7% reported harsh parenting, 24.8% reported caregiver stress, 12.2% reported caregiver symptoms of depression, 2.3% reported violence, and 2.6% reported substance use.

**Figure 1:**
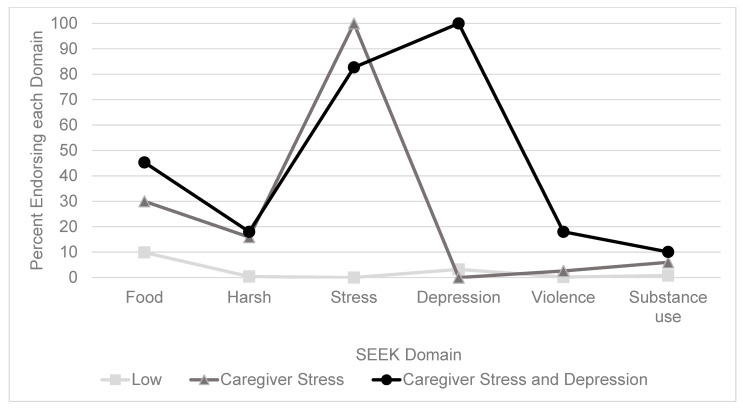
Latent class analysis three class solution for SEEK domains.

Heterogeneity in SEEK experiences was established; model fit indices and classification quality for 2–5 class solutions are presented in Table 2. SEEK classes were not associated with child sex or age (0–3 vs. 3–6 years of age). The 3-class model emerged as the best-fitting model based on an evaluation of fit indices, classification quality, and interpretability. The three identified classes of adverse experiences on the SEEK were the following: (1) low (L; *n* = 1034, 73%), (2) caregiver stress (CS; *n* = 233, 17%), and (3) caregiver stress and depression (CSD; *n* = 139, 10%) (Figure 1). Belonging to the L class was associated with a lower ACE-Q total and higher PACES total compared to the CS and CSD classes (Table 3).

In a post hoc exploratory analysis, we re-ran the multivariate GLM on a subsample of respondents who identified as the child’s parent (*n* = 916), excluding those who identified as a relative caregiver or nonbiological caregiver. In this analysis (Table 3), results were nearly identical to the original results with one additional significant group difference: ACE-Q total score was significantly greater for the CSD class compared to the CS class. Group differences in the PACES were the same in both samples.

## 4. Discussion

Among this group of young children attending well-child visits in an urban academic practice that predominantly serves Medicaid-eligible families, caregivers reported a range of adverse family experiences, including not only potential direct safety concerns (requests for poison control contact information and smoke alarms, children’s exposures to environmental tobacco smoke), but also health-related social determinants of health (food insecurity, caregiver depression, and caregiver substance use), and more general expressions of caregiver distress (desire for additional help with child and feeling under extreme stress). Both the setting—a university-based pediatric primary care resident continuity clinic—and the families served—those residing in a low-income urban community—are similar to the settings and populations assessed in prior studies of the implementation and performance of the SEEK instrument [40,47].

Despite these risks and concerns, the majority of families (73%) were empirically identified as a “low adversity” class/group, among whom relatively few (14%) screened positive in any category on the SEEK. Not surprisingly, these families with fewer contextual stressors or safety concerns reported significantly fewer adversities on the ACE-Q and significantly greater protective factors on the PACEs questionnaire.

The two remaining classes are notable for their shared inclusion of caregiver stress, with a greater proportion of families (30–45%) in these groups also indicating higher reports of food insecurity relative to the low class. This grouping is consistent with SEEK data generated by other primary care practices implementing this screener, where major parental stress and food insecurity were the most common risks endorsed by parents [35]. Given the presence of unmet material needs (food insecurity) among caregivers reporting stress in this and prior studies, we speculate that the caregiver stress and caregiver stress and depression classes may reflect, at least in part, the challenges faced by caregivers whose children may qualify for Medicaid but reside in households that earn too much for other means-based safety net services, such as the Special Nutrition Assistance Program (SNAP), Temporary Assistance for Needy Families (TANF), and Special Supplemental Nutrition Program for Women Infants and Children (WIC). Material hardship such as food insecurity is linked with adverse outcomes for children, and families who experience such hardship might be at increased risk for engaging in harsh parenting practices [48,49,50].

Caregiver experiences of stress, even in the absence of material hardship, have been linked with adverse outcomes for children in other ways. For example, parenting stress is higher among parents of children with emotional and behavioral challenges [51]. Parenting stress is also higher among caregivers of children with developmental delays or neurodevelopmental disorders [52], and among children with physical health problems [53]. For many caregivers, parenting stress stems from caring for children with high needs, although there is some bidirectional impact, with stressed caregivers engaging in more harsh parenting, which in turn can exacerbate child outcomes, including internalizing and externalizing problems. Importantly, we found that caregivers with elevated levels of caregiver stress reported fewer child protective factors relative to caregivers without elevated caregiver stress, suggesting that parents who experience a great deal of stress may need more support in being connected with potential protective factors.

It is notable that in analyses using the full sample, no differences in total ACEs or PACES were found between caregivers with elevated stress compared to caregivers with elevated levels of stress and depression. However, in post hoc exploratory analyses including only respondents who identified as the child’s parent, the caregiver stress and depression and caregiver stress classes still had higher average scores on the ACE-Q and lower average scores on the PACES than did the low-adversity class; additionally, the caregiver stress and depression class had higher ACE-Q scores than the caregiver stress (only) class. It appears from these data that the presence of parent stress with a depressed mood may have different meanings for children when experienced by parents as compared to other caregivers in terms of the total burden of adversity. For example, it may be that non-parent caregivers are not fully aware of children’s history of adverse experiences if they have not been the child’s primary caregiver for their entire life. Additionally, results may have differed in other ways depending on the distribution of caregiver type; for example, other latent classes may have emerged if the sample was made up entirely of biological parents or non-biological parents. The absence in this dataset of further demographic details, such as caregiver age, precludes further analyses to explore the meaning of these differences. For example, we cannot determine whether non-parent caregivers are older than parents, with accordingly greater life experience or different support networks to overcome the potential impact of the caregiver’s mood on the household environment. Additionally, the absence of detailed demographic data makes it challenging to determine if family experiences are similar across other demographic subgroups. Given what we know about social determinants of health, it stands to reason that associations among adversity and protective factors may differ based on family race or ethnicity, income, and other factors that are associated with greater instances of systemic and individual discrimination [18,20,22].

Other limitations of this study relate to the period during which data were collected; the earliest sets of screens were collected from a subset of clinicians piloting the new screener workflow within the practice prior to the onset of the COVID-19 pandemic public health emergency, with the expansion of screening to the full practice by spring 2020. These analyses did not account for secular pressures that might have influenced what needs families reported and in what combinations, such as changes in food or housing policies, or employment changes that might have influenced which caregivers reported extreme stress. Future analyses should explore whether the classes observed here (low adversity, caregiver stress, caregiver stress plus depression) are reproduced after the public health emergency ends. Finally, the specific profiles of adverse experiences identified through LCA in the present study may not generalize to other samples or measures of adversity. The large, representative nature of the sample does protect against this limitation to some extent; still, future work is needed to understand the experiences of caregiver stress and depression and the impact of their combination on the lives of young children. Replication of this work is also needed to explore factors that might affect class membership; we were only able to examine child sex and age in the present study due to the limited availability of demographic characteristics.

## 5. Conclusions

Among this population of US children obtaining well-child care in an urban pediatric primary care resident continuity clinic, three empirically derived classes of adverse experiences based on the SEEK screen were identified: (1) low adversity, (2) caregiver stress, and (3) caregiver stress and depression. Overall, belonging to the low-adversity class was associated with lower ACE-Q and higher PACES compared to the other two classes, while among biological parents, the caregiver stress and depression class was associated with the highest ACE-Q scores. Findings from the current study affirm that screening for specific types of adversity (rather than solely counts of adversities) is feasible within the context of well-child visits and that profiles of adversity (in addition to a review of individual items) can provide actionable information for clinicians and practices. In particular, experiences of stress and depression by caregivers of young children may be strong indicators of elevated risk factors and low levels of protective factors.

Future work is needed to better understand the meaning of the caregiver stress and caregiver stress and depression classes and what caregivers interpret as “extreme stress.” While other measures of adversity [24] might generate different profiles than observed in this practice using the SEEK, a better understanding of what parents mean to convey through their individual responses to the SEEK and related tools may inform practices’ selection of screeners and interventions to address caregivers’ needs. For example, pediatric practice-based interventions to meet the needs of caregivers with elevated levels of stress might focus on social risk interventions such as referral relationships with food banks [54], medical–legal partnerships [55], assistance completing applications for earned-income tax credits or other financial resources [56], or they might focus on parenting interventions to promote healthy relationships in the face of stress [57,58]. Practice-based interventions to meet the needs of parents with elevated levels of stress and depression might additionally consider strategies that promote resilience and scaffold protective factors around their families, for example, through the integration of peer support programming or dyadic or parent-focused screening and brief intervention and referral to treatment for their mental health concerns [59].

Indeed, in the US, emerging state and federal policies are incentivizing clinicians and practices to implement whole family or multigenerational interventions under an accountable healthcare framework that considers the broad set of social factors influencing physical emotional developmental, and relational health [60,61]. For example, the National Committee for Quality Assurance (NCQA) has recently added to its Healthcare Effectiveness Data Information System (HEDIS) measures that set a requirement for social risk screening and intervention applicable to patients of all ages [62]. The findings of the present study contribute to this larger conversation, reinforcing prior evidence that links caregiver well-being with their child’s experience of social risk [63,64].

In sum, pediatricians are well situated to survey early risk and provide guidance to caregivers and linkage to prevention programs and supports to benefit children and their families [2,13]. Based on the findings of our exploratory analysis among caregivers who identified as that child’s biological parent, pediatricians should particularly attend to the needs of biological parents reporting the combination of stress and depression on screeners such as the SEEK, as these families may face especially high levels of adversity and low levels of protective factors.

## Figures and Tables

**Table 1:** Sample characteristics and SEEK item endorsement.

	*n*	%
Child sex (Female)	718	51.4
Child age		
0–3 years old	725	55.9
3–6 years old	572	44.1
Relationship to respondent		
Parent	916	72.6
Other ^a^	346	27.4
SEEK item endorsement	*n*	%
Food insecurity	235	16.7
Worried food would run out	201	14.4
Food did not last	174	12.5
Harsh parenting	66	4.7
Child difficult to care for	42	3
Feel need to slap or hit child	29	2.1
Caregiver stress	348	24.8
Wish had more help with child	264	18.9
Extreme stress	182	13.1
Caregiver symptoms of depression	172	12.2
Down, depressed, or hopeless	130	9.3
Little interest or pleasure in doing things	115	8.3
Violence	33	2.3
You and partner fought a lot	31	2.2
Partner threatened you physically	6	0.4
Substance use	36	2.6
Four or more drinks in one day	29	2.1
Used an illegal drug or off-prescription drug	9	0.6
Additional items not included in class analysis		
Smoker in the home	230	16.5
Request for poison control number	278	20.2
Need smoke alarm in the home	100	7.2
Other things you would like help with today	39	2.9

^a^ Other includes aunt/uncle, grandparent, and unrelated caregiver.

**Table 2:** LCA model fit and classification quality.

Classes	AIC	BIC	LMR-LRT (Adjusted)	*p*-Value	BLRT	*p*-Value	Entropy	Posterior Probabilities
2	4597.45	4665.68	486.83	<0.001	−2533.94	<0.001	0.745	0.914–0.928
3	4580.19	4685.15	30.66	0.001	−2285.73	<0.001	0.955	0.935–0.994
4	4572.68	4714.39	21.10	0.067	−2270.09	<0.001	0.830	0.440–0.999
5	4575.86	4754.31	9.687	0.407	−2258.87	0.375	0.897	0.691–0.971

*Note.* AIC = Akaike information criteria. BIC = Bayesian information criteria. LMR-LRT = Lo–Mendel–Rubin likelihood ratio test (adjusted). BLRT = bootstrap likelihood ration test.

**Table 3:** Mean differences in ACE-Q and PACES total scores by SEEK class.

	Low (L)		Caregiver Stress (CS)		Caregiver Stress and Depression (CSD)					
	*m*	*sd*	*m*	*sd*	*m*	*sd*	*F*(df_1_, df_2_)	*p*-Value	Partial *η*^2^	Comparisons
Model 1: Full sample							39.48(4, 2476)	<0.001	0.06	
ACE-Q Total	0.38	0.93	1.17	1.78	1.44	1.75	69.30(2, 1239)	<0.001	0.10	CS, CSD > L
PACES Total	8.61	1.83	7.99	1.76	7.97	1.82	17.00(2, 1239)	<0.001	0.03	L > CS, CSD
Model 2: Parent subsample							32.58(4, 1626)	<0.001	0.07	
ACE-Q Total	0.37	0.97	1.09	1.39	1.60	1.80	59.05(2, 814)	<0.001	0.13	CS, CSD > L CSD > CS
PACES Total	8.61	1.74	7.98	1.80	8.00	1.75	12.30(2, 814)	<0.001	0.03	L > CS, CSD

## Data Availability

The data presented in this study are available on request from the corresponding author. The data are not publicly available due to the inclusion of protected health information.
